# The Relationship of Grief Reactions with Cognitive Emotion Regulation Strategies and Intolerance of Uncertainty in Adults

**DOI:** 10.3390/healthcare14193342

**Published:** 2026-10-08

**Authors:** Elif Yöyen, Rana Şan

**Affiliations:** 1 Department of Psychology, Faculty of Humanities and Social Sciences, Sakarya University, Sakarya 54050, Türkiye; 2 Institute of Social Sciences, Department of Psychology, Maltepe University, Maltepe, Istanbul 34857, Türkiye

**Keywords:** grief, cognitive emotion regulation, intolerance of uncertainty, loss, emotion regulation

## Abstract

**Background/Objectives**: Grief following the death of a loved one may be associated with cognitive emotion regulation strategies and responses to uncertainty. This study examined the relationships between grief, cognitive emotion regulation strategies, and intolerance of uncertainty among adults who had lost a loved one and whether grief differed according to demographic and loss-related characteristics. **Methods**: The sample comprised 304 adults aged 18–65 years who had experienced the death of a loved one 6–24 months previously. Data were collected using the Demographic Information Form, Grief Scale, Cognitive Emotion Regulation Questionnaire, and Intolerance of Uncertainty Scale. Independent-samples *t*-tests, one-way analysis of variance, Pearson correlation, and regression analyses were conducted. **Results**: After adjustment for multiple comparisons, grief was not significantly associated with age (*r* = −0.012) or time since loss (*r* = −0.053), but was positively associated with emotional closeness (*r* = 0.549, Holm-adjusted *p* < 0.001) and perceived social support (*r* = 0.202, Holm-adjusted *p* = 0.001). Grief was positively associated with self-blame (*r* = 0.445), rumination (*r* = 0.157), catastrophizing (*r* = 0.532), and blaming others (*r* = 0.348), and negatively associated with acceptance (*r* = −0.162), positive refocusing (*r* = −0.192), refocus on planning (*r* = −0.148), and positive reappraisal (*r* = −0.192); these associations remained significant after Holm adjustment. Putting into perspective was not significantly associated with grief after adjustment for multiple comparisons. Intolerance of uncertainty was positively associated with grief (*r* = 0.447, *p* < 0.001). In the hierarchical regression analysis, demographic and loss-related covariates accounted for 44.7% of the variance in grief scores (*R*^2^ = 0.447). Adding cognitive emotion regulation strategies accounted for an additional 18.2% (Δ*R*^2^ = 0.182, *p* < 0.001), and intolerance of uncertainty accounted for a further 1.1% (Δ*R*^2^ = 0.011, *p* = 0.020). The final model accounted for 64.0% of the variance (*R*^2^ = 0.640, adjusted *R*^2^ = 0.603). **Conclusions**: Specific cognitive emotion regulation strategies and greater intolerance of uncertainty were associated with grief levels, with self-blame, rumination, catastrophizing, and blaming others showing positive associations with grief, whereas acceptance, positive refocusing, refocus on planning, and positive reappraisal showed negative associations. These findings highlight the relevance of cognitive emotion regulation and intolerance of uncertainty to individual differences in grief; however, given the cross-sectional design, the observed associations should not be interpreted as establishing temporal or causal relationships.

## 1. Introduction

The loss of a loved one due to death is an inevitable and common experience that individuals may encounter at some point in their lives [1]. Grief has been regarded by many researchers as a natural, non-pathological process [2,3]. However, the way grief is experienced, its intensity, and the extent to which individuals readjust to life following a loss vary from person to person. Variables such as perceived social support following the loss, previous grief experiences, age, gender, economic status, the nature of the relationship with the deceased, and the manner of death may influence the extent to which an individual is affected by the loss. Therefore, understanding the grieving process requires consideration not only of the loss itself but also of individual and loss-related characteristics.

Grief can be defined as the entirety of physical, behavioral, emotional, and cognitive reactions that occur following the loss of a loved one [4]. According to Shear (2012), grief refers to the response to the experience of losing a loved one, whereas the grieving process involves accepting the finality and irreversibility of the loss and developing the capacity to resume one’s life [5]. Freud (1922) defined grief as a non-pathological response that emerges following the loss of a loved person or of a reality that is valued by the individual [2]. Similarly, Engel (1961) stated that although uncomplicated grief may involve shock and denial, intense emotional reactions, and somatic symptoms, individuals may gradually cope with the effects of the loss and readjust to their lives over time [6].

Although grief reactions vary among individuals, certain common physical, emotional, behavioral, and cognitive symptoms may be observed. Physical symptoms include a feeling of emptiness in the stomach, difficulty breathing, and bodily discomfort; emotional symptoms include shock, anger, guilt, fear, loneliness, and hopelessness; behavioral symptoms include changes in sleep and appetite, social withdrawal, and avoidance of situations that evoke reminders of the loss; and cognitive symptoms include denial, distractibility, forgetfulness, and distorted thoughts [7].

Various theoretical approaches have been developed to understand the grieving process. In Bowlby’s approach, grief is explained through processes of numbness or emotional blunting, yearning for the deceased, disorganization and despair, and reorganization. These phases are not separated by strict boundaries, and individuals may move back and forth between them [8]. Stroebe and Schut’s Dual Process Model conceptualizes coping with grief as an ongoing process between loss-oriented and restoration-oriented coping. In the loss-oriented process, individuals are preoccupied with thoughts, memories, and emotions related to the deceased, whereas in the restoration-oriented process, they attempt to cope with the roles, responsibilities, and life circumstances that have changed following the loss. Within this model, oscillation between these two processes is considered part of the adjustment to bereavement [9].

Worden explained the grieving process in terms of tasks to be accomplished and identified the acceptance of the reality of the loss, processing the physical and emotional pain caused by the loss, adjusting to life without the deceased, and reorganizing the emotional bond with the deceased while continuing with life as the fundamental tasks of grieving. Particularly in the third task, individuals are expected to undergo reorganization in internal, external, and spiritual domains of adjustment. Loss may affect not only individuals’ responsibilities in daily life but also their evaluations of themselves and the world [3].

When these theoretical approaches are considered together, grief appears not merely as an emotional response to the deceased but as a multidimensional process of adjustment encompassing individuals’ cognitive appraisals, emotions, behaviors, social roles, and expectations about the future. The intensity and course of grief may also be influenced by the identity of the deceased, the nature of the relationship, the manner of death, previous losses, individual characteristics, social support, and additional life difficulties encountered after the loss [3].

The manner of death is one of the variables that may influence the nature of grief. In anticipated losses, individuals have an opportunity to prepare for the loss and say goodbye to the person before death occurs, whereas sudden losses do not allow for such preparation. Therefore, grief may be more difficult to experience following sudden losses, and the recovery process may be prolonged [10]. Findings also suggest that individuals experiencing unexpected loss may be at greater risk of developing complicated grief [11]. In traumatic or unnatural deaths, cognitions such as self-blame and blaming others may emerge among bereaved individuals and may complicate the recovery process [12].

The identity of the deceased and the nature of the relationship with the deceased are also important in the grieving process. The loss of a spouse, child, or parent has been reported to be associated with higher levels of grief than other types of loss, and individuals who have lost a spouse or child have been identified as being at greater risk of complicated grief [13,14,15]. Findings regarding social support, however, are inconsistent. While some studies have shown that social support may facilitate adjustment by serving as a buffer during the grieving process, others have found no significant positive or negative effect [16,17]. These differences indicate that grief cannot be explained by a single factor and that individual characteristics and loss-related circumstances should be considered together.

### 1.1. Rationale and Significance of the Study

Although cognitive emotion regulation and intolerance of uncertainty have each been examined in relation to psychological adjustment, important gaps remain in the grief literature. Research on cognitive emotion regulation in bereavement has predominantly focused on specific strategies such as rumination and self-blame [18,19,20,21,22], while studies examining all nine cognitive emotion regulation strategies together remain limited. Similarly, research directly examining intolerance of uncertainty in relation to grief is scarce [23,24]. Moreover, these two cognitive domains have rarely been considered together while accounting for demographic and loss-related characteristics. Examining them within the same framework may therefore provide a more comprehensive understanding of individual differences in grief.

The present study addresses this gap in a sample of adults in Türkiye who had experienced the death of a loved one 6–24 months previously. Examining these relationships within this population also provides evidence from a cultural context that remains comparatively underrepresented in the existing literature.

### 1.2. Aim of the Study

The primary aim of this study was to examine the relationships between grief levels, cognitive emotion regulation strategies, and intolerance of uncertainty among adults in Türkiye who had lost a loved one due to death and whose loss had occurred at least 6 and no more than 24 months previously. The study also examined whether individuals’ grief levels differed according to demographic and loss-related characteristics, including gender, age, economic status, educational level, degree of kinship with the deceased, cause of death, frequency of contact with the deceased, emotional closeness, receipt of psychological/psychiatric support, experience of another loss, whether the loss was sudden, and perceived social support. In addition, the study aimed to examine the extent to which cognitive emotion regulation strategies and intolerance of uncertainty were independently associated with grief levels after accounting for theoretically relevant demographic and loss-related characteristics.

### 1.3. Research Questions and Hypotheses

The study was structured around three main research questions:

1. Do grief levels differ according to demographic and loss-related variables?
2. Is there a significant relationship between cognitive emotion regulation strategies and grief levels, and are cognitive emotion regulation strategies independently associated with grief levels after accounting for theoretically relevant demographic and loss-related characteristics?
3. Is there a significant relationship between intolerance of uncertainty and grief levels, and is intolerance of uncertainty independently associated with grief levels after accounting for theoretically relevant demographic and loss-related characteristics and cognitive emotion regulation strategies?

In line with these questions, cognitive emotion regulation strategies were expected to be significantly associated with grief levels and to account for additional variance in grief after adjustment for theoretically relevant demographic and loss-related characteristics. Intolerance of uncertainty was likewise expected to be significantly associated with grief levels and to account for additional variance after adjustment for demographic and loss-related characteristics and cognitive emotion regulation strategies.

The hypothesized associations of cognitive emotion regulation strategies and intolerance of uncertainty with grief, including their incremental associations in the hierarchical regression model, were treated as the primary analyses. Comparisons of grief across demographic and loss-related subgroups and analyses involving individual subscales or strategy-level associations were considered secondary or exploratory analyses and were interpreted with adjustment for multiple testing.

## 2. Theoretical Background

### 2.1. Cognitive Emotion Regulation and Grief

Emotion regulation refers to the set of processes that influence which emotions individuals experience, when they experience them, and how they experience and express these emotions [25]. This process may occur automatically or in a controlled manner, and consciously or unconsciously [26]. In Gross and Thompson’s approach, emotion regulation includes processes such as situation selection, situation modification, attentional deployment, cognitive change, and modulation of the resulting response.

Cognitive emotion regulation involves managing emotions that arise in response to a stressful life event through cognitive processes and adapting to new circumstances [27]. Cognitive emotion regulation strategies may contribute to increases or decreases in positive or negative emotions. Garnefski et al. (2001) conceptualized these strategies across nine dimensions: catastrophizing, rumination, self-blame, and blaming others are classified as maladaptive strategies, whereas acceptance, positive refocusing, refocus on planning, positive reappraisal, and putting into perspective are classified as adaptive strategies [28].

Adaptive and maladaptive strategies may be associated with different outcomes in terms of individuals’ psychological responses to stressful experiences. Maladaptive strategies have been reported to be more strongly associated with psychopathological symptoms, whereas adaptive strategies may be more functional in terms of psychological adjustment. The death of a loved one is also a stressful event that may be unfamiliar to the individual, create significant changes in life, and require readjustment. Therefore, cognitive emotion regulation strategies used following a loss are expected to be associated with grief reactions.

Rumination is one of the most extensively studied cognitive emotion regulation strategies in the grief literature. Rumination involves repeatedly processing negative emotions and thoughts. During the grieving process, rumination has been associated with the repeated mental processing of experiences related to the deceased, yearning, guilt, anger, or loneliness and has been suggested to complicate the grieving process [29]. Findings indicate that individuals who engage in rumination may experience more intense grief and exhibit higher levels of complicated grief symptoms [30,31].

Self-blame and blaming others are also cognitive strategies that become particularly relevant following unnatural losses. Weinberg (1994) reported that both self-blame and blaming others were more common following unnatural deaths and that, particularly when blaming others was accompanied by thoughts of revenge, recovery could be delayed [12]. In contrast, positive reappraisal may facilitate coping by assigning positive meaning to a negative experience. A study conducted with Indian and Japanese students reported that positive reappraisal may serve as a buffer for positive emotions during the grieving process [32].

A review of the literature indicates that studies examining cognitive emotion regulation in the grieving process have particularly focused on specific strategies such as rumination and self-blame [18,19,20,21,22]. In contrast, the limited number of studies evaluating the nine cognitive emotion regulation strategies defined by Garnefski et al. (2001) together in the context of grief highlights the importance of examining these strategies within a broader framework [28].

### 2.2. Intolerance of Uncertainty and Grief

Uncertainty is defined as the inability to fully predict expectations regarding the future and events that may occur in the future [33]. Intolerance of uncertainty refers to individuals’ cognitive, emotional, and behavioral responses to uncertainties concerning the future [34]. Individuals with high levels of intolerance of uncertainty may perceive uncertain situations as stressful, distressing, and unacceptable regardless of their likelihood of occurrence and may avoid uncertain situations or experience inhibition in the face of uncertainty [35].

Intolerance of uncertainty has also been suggested to have adverse effects on problem-solving processes. Individuals may perceive their circumstances as problematic even when there is no objectively negative situation or may engage in dysfunctional attempts to find solutions when confronted with an actual problem [34].

The loss of a loved one may confront individuals with new life circumstances that they have not previously experienced. Following a loss, individuals’ daily lives, self-evaluations, social roles, and plans for the future may change. During the grieving process, individuals must cope not only with the absence of the deceased but also with the new circumstances of life following the loss. Therefore, individuals who have difficulty tolerating uncertainty may experience greater difficulty adapting to these new circumstances.

The number of studies directly examining the relationship between intolerance of uncertainty and grief is limited. Existing research has primarily focused on the associations of intolerance of uncertainty with stress, anxiety, and generalized anxiety disorder. Nevertheless, the limited research in the field of grief points to the importance of this variable. Boelen (2010) reported a significant positive relationship between intolerance of uncertainty and complicated grief symptoms among individuals who had lost a loved one [36]. Boelen, Reijntjes, and Smid (2016) examined the prospective and inhibitory dimensions of intolerance of uncertainty in relation to symptoms of grief, post-traumatic stress, and depression and demonstrated that prospective intolerance of uncertainty predicted prolonged grief symptoms, whereas inhibitory intolerance of uncertainty was positively associated with symptoms of post-traumatic stress disorder and depression [37]. These findings indicate that intolerance of uncertainty is one of the variables that may be considered in understanding psychological symptoms experienced following a loss.

Loss due to death requires individuals not only to adapt to the absence of a loved one but also to cope with changing life circumstances following the loss. The role of the deceased in the individual’s life, changes in roles following the loss, and the reorganization of future plans may confront individuals with circumstances they have not previously encountered. From this perspective, examining the relationship between the uncertainty that emerges during the grieving process and individuals’ cognitive, emotional, and behavioral responses to uncertainty is important. This relationship may become particularly pronounced following unexpected losses because sudden losses do not allow individuals to prepare for the loss or begin the adjustment process before the loss occurs.

## 3. Materials and Methods

### 3.1. Study Design

A correlational survey design was used to examine the relationships between the grieving process, cognitive emotion regulation strategies, and intolerance of uncertainty. Survey research refers to quantitative research in which the same questions are systematically administered to a large number of participants and their responses are recorded [38]. The correlational survey design aims to determine the presence and degree of covariation between two or more variables. Given the cross-sectional and correlational nature of the study, the analyses were intended to quantify statistical associations among variables and were not designed to establish temporal or causal relationships.

### 3.2. Participants

The study sample consisted of 304 adults aged 18–65 years who had lost a loved one due to death and whose loss had occurred at least 6 and no more than 24 months previously. These age and time-since-loss criteria constituted the eligibility criteria for participation; no additional inclusion or exclusion criteria were applied. Participants were recruited in Türkiye using convenience sampling. Recruitment was conducted through both face-to-face and online channels.

Of the participants, 204 (67.1%) were women and 100 (32.9%) were men. The mean age was 34.64 ± 11.79 years. In terms of economic status, 56 participants (18.4%) reported a low economic status, 171 (56.3%) a moderate economic status, and 77 (25.3%) a high economic status. Regarding educational level, 10 participants (3.3%) were primary school graduates, 18 (5.9%) were secondary school graduates, 102 (33.6%) were high school graduates, 138 (45.4%) were university graduates, and 36 (11.8%) held a master’s or doctoral degree. Twenty-eight participants (9.2%) reported receiving psychiatric support during the grieving process, whereas 276 (90.8%) reported not receiving psychiatric support.

### 3.3. Data Collection Instruments

The Demographic Information Form was used to determine participants’ personal and loss-related characteristics, the Grief Scale to assess grief reactions, the Cognitive Emotion Regulation Questionnaire to assess cognitive emotion regulation strategies, and the Intolerance of Uncertainty Scale to assess levels of intolerance of uncertainty.

#### 3.3.1. Demographic Information Form

The Demographic Information Form was developed to assess participants’ demographic characteristics, such as gender, age, and economic status, as well as time since the loss, degree of kinship with the deceased, cause of death, emotional closeness to the deceased, frequency of contact with the deceased, whether psychological support was received during the grieving process, perceived social support from their social environment, and whether they had experienced the loss of another loved one within the previous 24 months. Because social support, previous grief experiences, gender, age, degree of kinship with the deceased, and cause of death have been considered factors that may play a role in the grieving process and post-traumatic growth following a loss, these variables were included in the Demographic Information Form [39]. Emotional closeness to the deceased was assessed using a single-item 10-point rating, with scores ranging from 1 to 10; higher scores represented greater perceived emotional closeness to the deceased. Perceived social support was likewise assessed using a single-item 10-point rating ranging from 1 to 10, with higher scores indicating greater perceived social support from the participant’s social environment.

#### 3.3.2. Grief Scale

The Grief Scale, adapted into Turkish by Balcı Çelik, was used to assess individuals’ grief reactions. The scale is a five-point Likert-type instrument consisting of 35 items assessing the physical, emotional, cognitive, and behavioral dimensions of grief. Total scores range from 35 to 175, with Items 11, 13, and 17 reverse-scored. Balcı Çelik (2006) reported that the internal consistency coefficients of the scale subdimensions ranged from 0.62 to 0.90, the Cronbach’s alpha coefficient for the total score was 0.96, and the test–retest reliability coefficient was 0.84 [7]. In the present study, the Cronbach’s alpha coefficient for the total Grief Scale score was 0.85. Cronbach’s alpha coefficients for the physical, emotional, cognitive, and behavioral subscales were 0.865, 0.659, 0.465, and 0.896, respectively.

#### 3.3.3. Cognitive Emotion Regulation Questionnaire

The Cognitive Emotion Regulation Questionnaire was developed by Garnefski, Kraaij, and Spinhoven (2001) to assess the cognitive emotion regulation strategies individuals use following specific stressful events [28] and was adapted into Turkish by Onat and Otrar. The five-point Likert-type scale consists of 36 items and nine subdimensions. These subdimensions are self-blame, rumination, acceptance, refocus on planning, positive reappraisal, putting into perspective, catastrophizing, blaming others, and positive refocusing [40]. Acceptance, refocus on planning, positive reappraisal, putting into perspective, and positive refocusing are classified as adaptive cognitive emotion regulation strategies, whereas self-blame, blaming others, catastrophizing, and rumination are classified as maladaptive cognitive emotion regulation strategies.

In the Turkish adaptation study, Onat and Otrar (2010) reported a Cronbach’s alpha coefficient of 0.78 for the scale [40]. In the present study, the Cronbach’s alpha coefficient for the Cognitive Emotion Regulation Questionnaire was 0.77. Cronbach’s alpha coefficients for self-blame, acceptance, rumination, positive refocusing, refocus on planning, positive reappraisal, putting into perspective, catastrophizing, and blaming others were 0.754, 0.724, 0.776, 0.615, 0.809, 0.823, 0.671, 0.756, and 0.843, respectively. For the hierarchical regression analysis, an adaptive cognitive emotion regulation composite score was calculated by summing the acceptance, positive refocusing, refocus on planning, positive reappraisal, and putting into perspective subscale scores, whereas a maladaptive composite score was calculated by summing the self-blame, rumination, catastrophizing, and blaming others subscale scores. In the present study, Cronbach’s alpha coefficients for the adaptive and maladaptive composite scores were 0.904 and 0.884, respectively. This grouping followed the adaptive–maladaptive classification of the CERQ strategies described above and was used to represent the two broader theoretically defined cognitive emotion regulation domains in the hierarchical regression model.

#### 3.3.4. Intolerance of Uncertainty Scale

The Intolerance of Uncertainty Scale was developed by Freeston, Rheaume, Letarte, Dugas, and Ladouceur (1994) to assess cognitive, behavioral, and emotional responses to uncertain situations [34] and was adapted into Turkish by Sarı and Dağ. While the original scale consists of 27 items, the Turkish version consists of 26 items and four factors. Higher scores on the scale indicate greater intolerance of uncertain situations. The four factors were defined as “Uncertainty is stressful and upsetting,” “Negative self-assessments about uncertainty,” “Disturbing thoughts about the uncertainty of the future,” and “Uncertainty interferes with action”. The internal consistency coefficient of the scale was reported as 0.93, and the test–retest reliability coefficient as 0.66. Cronbach’s alpha coefficients for the subdimensions were 0.88, 0.79, 0.79, and 0.79, respectively [41]. In the present study, the Cronbach’s alpha coefficient for the Intolerance of Uncertainty Scale was 0.87.

### 3.4. Data Collection Procedure and Ethics

Data collection commenced after ethics committee approval was obtained on 19 March 2021 and continued until June 2021. Participants were recruited using convenience sampling. Data were collected using a mixed-mode procedure, including both face-to-face paper-and-pencil administration and an online survey administered via Google Forms. Prior to analysis, responses were screened for completeness and eligibility according to the predefined age and time-since-loss criteria. Online responses were also screened for duplicate submissions, and only complete and eligible cases were retained for the final analyses. The final analytic sample consisted of 304 participants. No post hoc or observed power analysis was used to evaluate the adequacy of the sample size. Ethical approval for the study was obtained from the relevant ethics committee of Maltepe University under protocol number 2020/08-05.

Data were collected using both printed questionnaires administered face-to-face and an online questionnaire administered via Google Forms. Because data collection took place during the COVID-19 pandemic, the majority of responses were obtained online. The use of two data-collection modes represents a potential source of methodological heterogeneity and was considered when interpreting the findings. Participation in the study was voluntary. Written informed consent was obtained from participants who participated face-to-face, whereas digital informed consent was obtained from those who participated online. During online data collection, participant confidentiality was protected through anonymization procedures, and participants were informed that the information they provided would be used solely for research purposes. Data collection took approximately 15–20 min for each participant.

### 3.5. Statistical Analysis

Data obtained from the study were analyzed using IBM SPSS Statistics 22.0. Before conducting the analyses, the distributions of the total and subscale scores of the instruments used in the study were examined. Skewness and kurtosis values within ±1.5 were considered indicative of distributions sufficiently close to normality [42], and parametric statistical methods were therefore used.

Frequencies, percentages, means, and standard deviations were used to describe participants’ demographic and loss-related characteristics and the main study variables. Independent-samples *t*-tests were used to compare grief scores between two independent groups, whereas one-way analysis of variance (ANOVA) was used for comparisons involving more than two independent groups. When significant group differences were identified by ANOVA, Tukey’s post hoc test was applied to determine which groups accounted for the differences. Because some loss-related categories contained small and markedly unequal group sizes, the robustness of the corresponding group comparisons was additionally evaluated using Welch’s ANOVA. Where significant omnibus differences were identified, Games–Howell post hoc comparisons were used because this procedure does not require homogeneity of variances and is appropriate for unequal group sizes. Analyses involving extremely small subgroups were considered exploratory and were interpreted cautiously; categories were not combined in the absence of a clear conceptual justification.

Linear relationships between grief levels and age, time since the loss, emotional closeness to the deceased, perceived social support, cognitive emotion regulation strategies, and intolerance of uncertainty were examined using Pearson correlation analysis. Assumptions underlying the inferential analyses were examined before interpretation. Homogeneity of variance for group comparisons was evaluated using Levene’s test. When variance homogeneity was questionable, particularly in analyses involving small or unequal groups, Welch-based procedures were used as robust alternatives. For Pearson correlations, linearity and the potential influence of individual observations were examined; rank-based sensitivity analyses were conducted when departures from linearity were indicated.

A hierarchical multiple linear regression analysis was conducted to examine the incremental cross-sectional associations of cognitive emotion regulation strategies and intolerance of uncertainty with grief. Covariates were selected a priori on theoretical grounds based on their conceptual relevance to grief and the loss experience, rather than solely on the basis of statistical significance in univariate analyses. Accordingly, age, gender, emotional closeness to the deceased, perceived social support, sudden loss, experience of another loss, psychiatric support, frequency of contact with the deceased, and degree of kinship were entered in the first block. These covariates were included to account for potentially relevant demographic and loss-related characteristics when estimating the independent associations of cognitive emotion regulation strategies and intolerance of uncertainty with grief. Adaptive and maladaptive cognitive emotion regulation strategies were entered in the second block, followed by intolerance of uncertainty in the third block. Changes in explained variance (Δ*R*^2^) were evaluated at each step. Regression diagnostics included assessment of linearity, residual independence, homoscedasticity, multicollinearity, and potentially influential observations. Multicollinearity was evaluated using variance inflation factors (VIFs), residual independence using the Durbin–Watson statistic, heteroscedasticity using the Breusch–Pagan test, and influential observations using Cook’s distance and studentized residuals. Because the Breusch–Pagan test indicated heteroscedasticity, HC3 heteroscedasticity-consistent robust standard errors were used for inference in the final regression model. Effect sizes were reported to complement significance testing. Hedges’ *g* with 95% confidence intervals was used for two-group comparisons, whereas ω^2^ was reported for comparisons involving more than two groups. Pearson correlation coefficients were accompanied by conventional two-sided 95% confidence intervals calculated using Fisher’s r-to-z transformation; these confidence intervals were not multiplicity-adjusted. Unstandardized regression coefficients were reported with 95% confidence intervals.

To address the risk of Type I error arising from multiple comparisons, the Holm procedure was applied to secondary and exploratory analyses within conceptually defined families of related tests. These families included comparisons across grief dimensions for demographic and loss-related characteristics, correlations of grief with demographic and loss-related continuous variables, individual cognitive emotion regulation strategies, and dimensions of intolerance of uncertainty. The hierarchical regression analyses addressing the primary hypotheses were not included in these multiplicity adjustments. Statistical significance for secondary and exploratory analyses was evaluated using Holm-adjusted *p* values, while unadjusted *p* values were also retained to facilitate interpretation of the original analyses. All statistical tests were two-tailed, with a 95% confidence level and statistical significance set at *p* < 0.05. Exact *p* values were reported whenever possible, whereas very small values were reported as *p* < 0.001.

No a priori power analysis was conducted to determine the sample size, and retrospective observed-power analysis was not used. The sample size was determined by the number of eligible participants who completed the study during the predefined data-collection period.

## 4. Results

### 4.1. Demographic and Loss-Related Characteristics of the Participants

A total of 304 adults participated in the study. The mean age of the participants was 34.64 ± 11.79 years; 204 (67.1%) were women and 100 (32.9%) were men. Of the participants, 56.3% reported a moderate economic status, and 45.4% were university graduates. The proportion of participants who reported receiving psychiatric support was 9.2%.

The mean time since the loss was 13.91 ± 5.77 months, the mean emotional closeness to the deceased was 7.31 ± 2.38, and the mean perceived social support was 6.01 ± 2.61. Of the participants, 21.7% had lost a first-degree relative, 32.2% a second-degree relative, and 30.3% a third-degree relative. Illness accounted for 83.9% of the causes of death. In addition, 22.0% of the participants had experienced another loss within the previous 24 months, whereas 34.5% reported that the loss had been sudden. The results are presented in Table 1.

### 4.2. Descriptive Findings for the Study Measures

The mean total Grief Scale score was 76.34 ± 19.88. The mean scores for the physical, emotional, cognitive, and behavioral grief subscales were 10.47 ± 4.55, 20.80 ± 5.87, 27.02 ± 5.40, and 18.04 ± 7.57, respectively. The mean total Cognitive Emotion Regulation Questionnaire score was 107.12 ± 18.60, whereas the mean total Intolerance of Uncertainty Scale score was 78.45 ± 22.01. The findings are presented in Table 2.

### 4.3. Grief Reactions According to Demographic Characteristics

Comparisons of grief reactions according to gender, economic status, and educational level were treated as secondary analyses, and Holm adjustment was applied separately within each demographic characteristic across the four grief dimensions. Women had significantly higher cognitive grief scores than men (M = 27.56, SD = 5.49 vs. M = 25.92, SD = 5.05), and this difference remained statistically significant after Holm adjustment (raw *p* = 0.012, Holm-adjusted *p* = 0.049; Hedges’ g = 0.31, 95% CI [0.07, 0.55]). No statistically significant gender differences were observed for physical, emotional, or behavioral grief. Grief scores also did not differ significantly according to economic status or educational level after adjustment for multiple comparisons. Unadjusted and Holm-adjusted *p* values, together with effect sizes and 95% confidence intervals, are presented in Table 3.

### 4.4. Grief Reactions According to Loss-Related Characteristics

Comparisons according to loss-related characteristics were treated as secondary analyses, and Holm adjustment was applied separately within each characteristic across the four grief dimensions. Given the small and unequal subgroup sizes, robust analyses were conducted using Welch’s ANOVA. Degree of kinship was significantly associated with physical, emotional, cognitive, and behavioral grief in the Welch analyses, and these associations remained statistically significant after adjustment for multiple comparisons. Games–Howell post hoc comparisons generally indicated lower grief scores among participants who had lost a third-degree relative than among several of the other kinship groups. Comparisons involving the very small spouse subgroup were considered exploratory and were interpreted cautiously. In contrast, Welch analyses showed no significant differences according to cause of death in physical, emotional, cognitive, or behavioral grief. Because some cause-of-death categories contained very few participants, these analyses were considered exploratory. The results are presented in Table 4.

For frequency of contact, significant differences were observed across all four grief dimensions, and all remained significant after Holm correction (all Holm-adjusted *p* < 0.001). Participants receiving psychiatric support had significantly higher physical, emotional, cognitive, and behavioral grief scores than those not receiving psychiatric support, with all four comparisons remaining significant after Holm correction (all Holm-adjusted *p* < 0.001). Participants who had experienced another loss also had significantly higher scores across all four grief dimensions; these differences remained significant after Holm correction (physical: Holm-adjusted *p* < 0.001; emotional: *p* = 0.015; cognitive: *p* = 0.019; behavioral: *p* = 0.019). Similarly, participants who had experienced a sudden loss had significantly higher scores across all four grief dimensions, and all differences remained significant after Holm correction (physical: *p* = 0.012; emotional: *p* = 0.006; cognitive: *p* < 0.001; behavioral: *p* = 0.009) (Table 5).

### 4.5. Relationships of Grief with Age, Time Since Loss, Emotional Closeness, and Social Support

After Holm adjustment across the four correlations, total grief was not significantly associated with age (*r* = −0.012, Holm-adjusted *p* = 0.835) or time since the loss (*r* = −0.053, Holm-adjusted *p* = 0.714). In contrast, grief was positively associated with emotional closeness to the deceased (*r* = 0.549, Holm-adjusted *p* < 0.001) and perceived social support (*r* = 0.202, Holm-adjusted *p* = 0.001). The results are presented in Table 6.

### 4.6. Relationships Between Grief and Cognitive Emotion Regulation Strategies

After Holm adjustment across the nine individual cognitive emotion regulation strategies, total grief was positively associated with self-blame (*r* = 0.445, Holm-adjusted *p* < 0.001), rumination (*r* = 0.157, Holm-adjusted *p* = 0.019), catastrophizing (*r* = 0.532, Holm-adjusted *p* < 0.001), and blaming others (*r* = 0.348, Holm-adjusted *p* < 0.001). Significant negative associations were observed with acceptance (*r* = −0.162, Holm-adjusted *p* = 0.019), positive refocusing (*r* = −0.192, Holm-adjusted *p* = 0.005), refocus on planning (*r* = −0.148, Holm-adjusted *p* = 0.020), and positive reappraisal (*r* = −0.192, Holm-adjusted *p* = 0.005). The association with putting into perspective was not statistically significant after correction for multiple comparisons (*r* = 0.044, Holm-adjusted *p* = 0.445). The total cognitive emotion regulation score was positively correlated with grief (*r* = 0.152, unadjusted *p* = 0.008); this total-score correlation was not included in the nine-strategy Holm family. The results are presented in Table 7.

### 4.7. Relationships Between Grief and Intolerance of Uncertainty

Total grief was significantly and positively associated with all four dimensions of intolerance of uncertainty. After Holm adjustment, significant positive associations remained for uncertainty being stressful and upsetting (*r* = 0.380, Holm-adjusted *p* < 0.001), negative self-assessments about uncertainty (*r* = 0.447, Holm-adjusted *p* < 0.001), disturbing thoughts about the uncertainty of the future (*r* = 0.434, Holm-adjusted *p* < 0.001), and uncertainty interfering with action (*r* = 0.359, Holm-adjusted *p* < 0.001). Total intolerance of uncertainty was also positively associated with total grief (*r* = 0.447, unadjusted *p* < 0.001). The total-score correlation was not included in the four-dimension Holm family. The results are presented in Table 8.

### 4.8. Cross-Sectional Associations of Cognitive Emotion Regulation Strategies and Intolerance of Uncertainty with Grief

A hierarchical multiple linear regression analysis was conducted to examine the incremental cross-sectional associations of cognitive emotion regulation strategies and intolerance of uncertainty with grief. In the first block, theoretically selected demographic and loss-related covariates were entered, including age, gender, emotional closeness to the deceased, perceived social support, sudden loss, experience of another loss, psychiatric support, frequency of contact with the deceased, and degree of kinship. The first model was statistically significant, F = 9.53, *p* < 0.001, accounting for 44.7% of the variance in total grief scores (*R*^2^ = 0.447, adjusted *R*^2^ = 0.400). Thus, the subsequent increments in explained variance associated with cognitive emotion regulation strategies and intolerance of uncertainty were estimated after accounting for these demographic and loss-related covariates. Diagnostic analyses indicated no problematic multicollinearity, with VIF values ranging from 1.15 to 3.24. The Durbin–Watson statistic was 1.25. The Breusch–Pagan test indicated heteroscedasticity (*p* < 0.001); therefore, HC3 heteroscedasticity-consistent robust standard errors were used for coefficient-level inference. The maximum Cook’s distance was 0.105, indicating that no single observation exerted extreme influence on the fitted model. Examination of the functional form did not indicate a statistically significant departure from linearity.

In the second block, adaptive and maladaptive cognitive emotion regulation strategies were added to the model. Their addition significantly increased the explained variance by 18.2% (Δ*R*^2^ = 0.182, ΔF = 42.97, *p* < 0.001). The second model accounted for 62.9% of the variance in grief scores (*R*^2^ = 0.629, adjusted *R*^2^ = 0.593).

In the third and final block, intolerance of uncertainty was added. Intolerance of uncertainty accounted for an additional 1.1% of the variance beyond the demographic and loss-related covariates and cognitive emotion regulation strategies (Δ*R*^2^ = 0.011, ΔF = 5.48, *p* = 0.020). The final model was statistically significant, F = 17.21, *p* < 0.001, and accounted for 64.0% of the variance in grief scores (*R*^2^ = 0.640, adjusted *R*^2^ = 0.603). Thus, intolerance of uncertainty showed a statistically significant incremental association with grief after accounting for cognitive emotion regulation strategies and the theoretically selected covariates. The results are presented in Table 9.

## 5. Discussion

In this study, the relationships between grief reactions and demographic and loss-related characteristics, as well as the relationships of cognitive emotion regulation strategies and intolerance of uncertainty with grief, were examined among adults who had lost a loved one due to death. Overall, the findings indicate that grief is associated not only with the occurrence of the loss itself but also with the nature of the relationship with the deceased, characteristics of the loss, and the cognitive processes individuals use following the loss. In particular, maladaptive cognitive emotion regulation strategies and intolerance of uncertainty were associated with higher levels of grief.

### 5.1. Demographic and Loss-Related Characteristics and Grief

With regard to gender, no significant differences were found in physical, emotional, or behavioral grief symptoms after adjustment for multiple comparisons. However, women had significantly higher cognitive grief scores than men, and this difference remained statistically significant after Holm adjustment (Holm-adjusted *p* = 0.049). The effect size for this difference was small (Hedges’ g = 0.31), suggesting that the magnitude of the gender difference in cognitive grief was modest. Therefore, the findings do not provide sufficient evidence for gender differences across the dimensions of grief examined in the present study.

No significant differences were found in the physical, emotional, cognitive, or behavioral dimensions of grief according to economic status or educational level. Thus, the expectation that economic status would be negatively associated with grief reactions was not supported. Similarly, grief reactions did not differ significantly across educational levels. The unequal distribution of participants across the groups for these variables should be considered when evaluating the absence of significant group differences.

The degree of kinship with the deceased was significantly associated with all dimensions of grief. Participants who had lost a third-degree relative had lower physical, emotional, cognitive, and behavioral grief scores than the other groups. The identity of the deceased and the role of that person in the individual’s life are among the important factors that may influence the course of grief [43]. This finding indicates that the nature of the relationship with the deceased is important in determining the intensity of the grief experience.

Grief levels increased as emotional closeness increased (*r* = 0.549, Holm-adjusted *p* < 0.001). Similarly, participants who had been in daily contact with the deceased had higher physical, emotional, cognitive, and behavioral grief symptoms than those in the other contact-frequency groups. Köroğlu (2021) suggested that spending more time with the deceased may strengthen the bond between individuals [44]. Within this framework, the findings concerning emotional closeness, frequency of contact, and the identity of the deceased demonstrate a consistent pattern.

With regard to cause of death, no significant differences were observed in physical, emotional, cognitive, or behavioral grief symptoms after adjustment for multiple comparisons. Thus, the present findings do not provide sufficient evidence that grief dimensions differed according to cause of death. However, the subgroup analyses for degree of kinship and cause of death should be interpreted cautiously because some categories contained very small and markedly unequal numbers of participants. Although robust Welch analyses and, where applicable, Games–Howell post hoc comparisons were used to reduce the influence of unequal group sizes and variance heterogeneity, findings involving the smallest subgroups were considered exploratory and require confirmation in samples with more balanced subgroup distributions.

The finding that participants who experienced a sudden loss had significantly higher physical, emotional, cognitive, and behavioral grief symptoms than those who did not experience a sudden loss further supports this pattern. In anticipated losses, having time to say goodbye to the deceased and prepare for the loss has been suggested to facilitate the grieving process [10]. In contrast, findings indicate that the risk of developing complicated grief may be higher following sudden losses [11]. Therefore, the present findings regarding sudden loss are broadly consistent with previous literature; however, the current study assessed general grief reactions rather than clinically diagnosed prolonged or complicated grief.

Participants who had experienced another loss within the previous 24 months showed significantly higher physical, emotional, cognitive, and behavioral grief scores than those who had not experienced another loss, and all four differences remained statistically significant after adjustment for multiple comparisons. Thus, experiencing another recent loss was associated with higher grief reactions across all four dimensions examined in the present study.

Participants who received psychiatric support had higher scores across all grief subscales than those who did not receive such support. This finding should not be interpreted as indicating that support increases grief symptoms; the present study examined the relationship between receiving psychiatric support and grief symptoms. In the study by Schut et al. (2001), psychological support had neither a positive nor a negative effect among individuals experiencing normal grief, whereas positive effects of psychotherapy were reported among individuals exhibiting complicated grief reactions [45].

No significant relationships were found between total grief and either age or time since the loss. Longitudinal studies have reported that grief symptoms may decrease over time following a loss. van der Houwen et al. (2010) reported a decrease in grief reactions and an improvement in mental health over a six-month period [46]. However, the effect of time may differ in cases of complicated grief [47]. This comparison should likewise be interpreted cautiously because the present study did not assess a clinical grief disorder. Murphy et al. (1999) also demonstrated that the course of symptoms over time may vary across repeated assessments following the loss of a child [48]. The restriction of time since loss to 6–24 months in the present study should also be considered when evaluating this finding.

Perceived social support was positively associated with total grief (*r* = 0.202, Holm-adjusted *p* = 0.001), contrary to the expected negative association. However, this finding should be interpreted cautiously. Given the cross-sectional design, it cannot be concluded that greater social support contributes to greater grief. Individuals experiencing more intense grief may seek or receive more support, and the single-item measure used in the present study did not assess the quality, type, or adequacy of support. Therefore, the positive association observed here should not be interpreted as evidence that social support adversely affects the grieving process.

The findings should also be considered within the cultural context of Türkiye. Grief is embedded in social, familial, religious, and cultural practices, and previous research conducted in Türkiye has highlighted the role of death rituals and religious beliefs in bereavement experiences [1]. Accordingly, the associations observed in the present sample, particularly those involving emotional closeness, kinship, frequency of contact, and perceived social support, should be interpreted within this sociocultural context rather than assumed to generalize directly to populations with different cultural practices.

The timing of data collection should also be considered when interpreting the findings. Data were collected between March and June 2021, during the COVID-19 pandemic, and the majority of participants completed the study online. The pandemic context may have affected bereavement experiences through disruptions in social interaction, mourning practices, and access to interpersonal support. Because the present study did not directly assess pandemic-specific experiences, the extent to which the COVID-19 context influenced grief levels or the observed associations cannot be determined. The findings should therefore be interpreted with this contextual limitation in mind.

### 5.2. Cognitive Emotion Regulation Strategies and Grief

One of the main findings of the study was that total grief was positively associated with self-blame, catastrophizing, and blaming others after adjustment for multiple comparisons. Rumination also showed a significant positive association with grief, and this association remained statistically significant after Holm adjustment.

Consistent with this finding, the hierarchical regression analysis showed that cognitive emotion regulation strategies accounted for an additional 18.2% of the variance in grief scores after adjustment for theoretically relevant demographic and loss-related characteristics. Greater use of maladaptive cognitive emotion regulation strategies was associated with higher grief levels, whereas greater use of adaptive cognitive emotion regulation strategies was associated with lower grief levels.

Rumination has been associated with grief-related difficulties in previous studies [19,29,30,49]. Consistent with this literature, rumination showed a significant positive association with grief in the present study, and this association remained statistically significant after adjustment for multiple comparisons. The positive relationships of self-blame and blaming others with grief are also consistent with the existing literature. These strategies have been reported to occur more frequently, particularly following sudden and violent losses [12]. Blaming others may make forgiveness more difficult following a negative event and may maintain the individual’s focus on past experiences, thereby complicating the grieving process.

One of the strongest positive relationships with grief was found for catastrophizing (*r* = 0.532). Catastrophizing is associated with interpreting future events negatively while disregarding other possible outcomes [50]. Following a loss, individuals who engage in catastrophizing may make more hopeless and anxious evaluations of their future, and thoughts that they will be unable to recover from the loss or that their lives will not go well may complicate the grieving process.

In contrast, significant negative relationships were found between grief and positive refocusing and positive reappraisal. It has been reported that assigning positive meaning to a negative life experience through positive reappraisal and positive refocusing may help individuals cope with anxiety-provoking situations and that functional cognitive emotion regulation strategies may serve as a buffer during the grieving process [32]. In this respect, the present finding is consistent with the literature.

However, the expected relationships did not emerge for all cognitive emotion regulation strategies. No significant relationship was found between total grief and putting into perspective after adjustment for multiple comparisons. In contrast, acceptance and refocus on planning showed significant negative associations with grief after Holm correction. These findings indicate that the relationship between cognitive emotion regulation strategies and grief may differ at the level of individual strategies rather than reflecting a uniform pattern across all adaptive strategies.

Taken together, the correlation findings indicate that individual cognitive emotion regulation strategies showed distinct associations with grief. Self-blame, rumination, catastrophizing, and blaming others were positively associated with grief, whereas acceptance, positive refocusing, refocus on planning, and positive reappraisal were negatively associated with grief after adjustment for multiple comparisons. Putting into perspective was not significantly associated with grief. The hierarchical regression findings further indicated that cognitive emotion regulation strategies accounted for additional variance in grief after demographic and loss-related characteristics were taken into account. These findings suggest that cognitive emotion regulation strategies are relevant to individual differences in grief; however, given the cross-sectional design, the temporal direction of these associations cannot be established.

### 5.3. Intolerance of Uncertainty and Grief

Another main finding of the study was that intolerance of uncertainty was positively associated with grief. All subdimensions of intolerance of uncertainty were positively associated with the total grief score. In the hierarchical regression analysis, intolerance of uncertainty accounted for an additional 1.1% of the variance in grief scores after demographic and loss-related characteristics and cognitive emotion regulation strategies had been taken into account (Δ*R*^2^ = 0.011, *p* = 0.020). Thus, intolerance of uncertainty showed a statistically significant incremental association with grief beyond the variables entered in the preceding blocks.

Intolerance of uncertainty is associated with perceiving uncertain situations as threatening and holding negative core beliefs about uncertainty [51]. Individuals with high levels of intolerance of uncertainty may perceive uncertain situations as stressful and unacceptable regardless of their likelihood of occurrence and may exhibit avoidance or inhibition in such situations [35].

From this perspective, loss due to death is particularly important in relation to intolerance of uncertainty. The loss of a loved one may alter individuals’ evaluations of their identity, roles, goals, and plans for the future and may confront them with new uncertainties. The ability to tolerate uncertainty may help individuals reevaluate their self-perceptions, roles, and goals following a loss and confront painful thoughts, emotions, and memories associated with separation. In contrast, individuals with high levels of intolerance of uncertainty may experience greater difficulty changing their views of their new circumstances and the future [52].

The present finding is also consistent with the limited literature directly examining the relationship between grief and intolerance of uncertainty. Boelen (2010) reported a significant positive relationship between intolerance of uncertainty and complicated grief symptoms [36]. However, the Grief Scale used in the present study assesses general grief reactions and does not provide a diagnosis of prolonged grief disorder or complicated grief. Therefore, comparisons with studies examining clinical or prolonged/complicated grief should be interpreted as conceptual comparisons rather than evidence that the present participants met diagnostic criteria for prolonged grief disorder. Boelen, Reijntjes, and Smid (2016) reported a prospective association between intolerance of uncertainty and prolonged grief symptoms [37]. These findings concern prolonged grief symptoms and should not be interpreted as directly equivalent to the general grief reactions assessed in the present study. In addition, individuals with high levels of intolerance of uncertainty have been reported to be more negatively affected by stressful and anxiety-provoking situations and to experience difficulty adapting to unexpected circumstances. Psychological responses to acute evaluative stress have also been documented in other contexts; for example, Ciaccioni et al. reported psychological and physiological changes associated with a demanding evaluative situation. However, such acute performance-related stress is conceptually distinct from bereavement, and these findings should therefore be considered only as broader evidence that psychologically demanding situations may elicit measurable stress responses [53].

Given the limited number of studies directly examining intolerance of uncertainty and grief, this finding represents one of the important results of the present study. When the higher scores across all grief dimensions among participants who experienced a sudden loss are considered together with the positive association between intolerance of uncertainty and grief, uncertainties regarding changing life circumstances and the future following a loss appear to be relevant to the grief experience. Findings indicating that intolerance of uncertainty is associated with the development and maintenance of emotional problems following stressful life events also support this interpretation [54]. Given the cross-sectional design of the present study, the direction of these associations cannot be established. Although cognitive emotion regulation strategies and intolerance of uncertainty were associated with grief levels, reverse or reciprocal relationships are also plausible. For example, more intense grief may be associated with greater rumination, catastrophizing, or difficulty tolerating uncertainty. Therefore, the observed associations should not be interpreted as evidence that these cognitive characteristics temporally or causally influence grief.

### 5.4. General Evaluation

When the findings of the study are considered together, three main patterns emerge. First, grief reactions appear to be more clearly associated with characteristics of the relationship with the deceased and the nature of the loss than with economic status or educational level. Emotional closeness, frequency of contact, degree of kinship with the deceased, and the suddenness of the loss were associated with grief symptoms; experiencing another loss within the previous 24 months was likewise associated with significantly higher grief scores across all four dimensions after adjustment for multiple comparisons. These findings are consistent with the literature regarding the importance of emotional closeness, frequency of contact, and the identity of the deceased in the grieving process.

Second, individual cognitive emotion regulation strategies showed distinct associations with grief levels. Self-blame, rumination, catastrophizing, and blaming others were positively associated with grief after adjustment for multiple comparisons, whereas acceptance, positive refocusing, refocus on planning, and positive reappraisal were negatively associated with grief. Putting into perspective was not significantly associated with grief. In the hierarchical regression analysis, cognitive emotion regulation strategies accounted for an additional 18.2% of the variance in grief scores after adjustment for theoretically relevant demographic and loss-related characteristics. Greater use of maladaptive cognitive emotion regulation strategies was associated with higher grief levels, whereas greater use of adaptive cognitive emotion regulation strategies was associated with lower grief levels.

Third, intolerance of uncertainty was associated with higher levels of grief. In the hierarchical regression analysis, intolerance of uncertainty accounted for an additional 1.1% of the variance in grief scores after demographic and loss-related characteristics and cognitive emotion regulation strategies had been taken into account. This incremental association suggests that intolerance of uncertainty is relevant to individual differences in grief beyond the variables entered in the preceding blocks.

Taken together, these results highlight the importance of considering cognitive emotion regulation strategies and intolerance of uncertainty together in understanding the grieving process. The fact that studies examining cognitive emotion regulation and grief have predominantly focused on specific strategies such as rumination and self-blame, together with the limited number of studies directly examining intolerance of uncertainty and grief, underscores the importance of examining these two groups of variables within the same framework.

## 6. Strengths and Limitations

A strength of the present study is the simultaneous examination of cognitive emotion regulation strategies and intolerance of uncertainty together with a broad set of demographic and loss-related characteristics. The use of adjusted multivariable analyses and correction for multiple comparisons also allowed the findings to be evaluated more conservatively.

Several limitations should be considered. First, the cross-sectional design precludes conclusions regarding temporal or causal relationships. Second, convenience sampling and recruitment from Türkiye limit the generalizability of the findings to other populations and cultural contexts. Third, the study relied on self-report measures, which may introduce common-method variance and response bias. In addition, the cognitive subscale of the Grief Scale showed low internal consistency in the present sample (Cronbach’s α = 0.465). Accordingly, findings involving the cognitive grief subscale should be interpreted cautiously because measurement unreliability may limit the precision and stability of associations involving this subscale. The restriction of time since loss to 6–24 months also limits generalization beyond this period. Some loss-related subgroups were small and markedly unequal in size; therefore, these subgroup findings should be considered exploratory. In addition, the use of both online and paper-and-pencil data collection may have introduced mode-related heterogeneity. Moreover, because data were collected during the COVID-19 pandemic, pandemic-related circumstances may have influenced participants’ bereavement experiences and responses. Although multiple-testing procedures and theoretically selected covariates were used, residual confounding and Type I error cannot be completely excluded. Finally, the cross-sectional design does not allow the temporal direction of the observed associations to be established.

### Future Research Directions

Future studies should employ longitudinal designs to clarify the temporal relationships among grief, cognitive emotion regulation strategies, and intolerance of uncertainty. Research using larger, more representative, and culturally diverse samples would also help determine the generalizability of the present findings. Further studies may examine these relationships across different types of loss and time periods following bereavement and may use multi-method assessments to reduce reliance on self-report measures. In addition, future research could investigate whether cognitive emotion regulation strategies and intolerance of uncertainty represent potentially relevant targets for psychological interventions designed to support bereaved individuals.

## 7. Conclusions

This study examined the relationships of grief reactions with cognitive emotion regulation strategies, intolerance of uncertainty, and various demographic and loss-related characteristics among adults who had lost a loved one due to death.

The main findings of the study showed that individual cognitive emotion regulation strategies were significantly associated with grief levels. Self-blame, rumination, catastrophizing, and blaming others were positively associated with grief after adjustment for multiple comparisons, whereas acceptance, positive refocusing, refocus on planning, and positive reappraisal were negatively associated with grief. Putting into perspective was not significantly associated with grief. In the hierarchical regression analysis, cognitive emotion regulation strategies accounted for an additional 18.2% of the variance in grief scores after adjustment for theoretically relevant demographic and loss-related characteristics. Greater use of maladaptive cognitive emotion regulation strategies was associated with higher grief levels, whereas greater use of adaptive cognitive emotion regulation strategies was associated with lower grief levels.

Intolerance of uncertainty was also significantly and positively associated with grief. All subdimensions of intolerance of uncertainty were positively associated with the total grief score. In the hierarchical regression analysis, intolerance of uncertainty accounted for an additional 1.1% of the variance in grief scores after demographic and loss-related characteristics and cognitive emotion regulation strategies had been taken into account. These findings indicate that intolerance of uncertainty is relevant to individual differences in grief. The study also found that grief symptoms were associated with degree of kinship with the deceased, frequency of contact, emotional closeness, and the suddenness of the loss. Experiencing another loss within the previous 24 months was associated with significantly higher physical, emotional, cognitive, and behavioral grief scores after adjustment for multiple comparisons. In contrast, no significant relationships were found between total grief and either age or time since the loss. No significant differences were found in the grief subdimensions according to economic status or educational level.

In conclusion, the findings highlight the relevance of considering cognitive emotion regulation strategies and intolerance of uncertainty together when examining individual differences in grief. Given that previous research on cognitive emotion regulation and grief has predominantly focused on specific strategies and that research directly examining intolerance of uncertainty in relation to grief remains limited, evaluating these psychological variables within the same study contributes to the existing literature. However, given the cross-sectional design, convenience sampling, self-report methodology, and other limitations described above, the findings should be interpreted cautiously as statistical associations rather than evidence of temporal or causal relationships. Longitudinal studies with more representative and culturally diverse samples are needed to clarify the direction and generalizability of these associations.

## Figures and Tables

**Table 1:** Demographic and Loss-Related Characteristics of Participants.

Variable	Category/Statistic	Value
Age, years	M (SD)	34.64 (11.79)
Gender	Women	204 (67.1%)
	Men	100 (32.9%)
Economic status	Low	56 (18.4%)
	Moderate	171 (56.3%)
	High	77 (25.3%)
Education level	Primary school	10 (3.3%)
	Secondary school	18 (5.9%)
	High school	102 (33.6%)
	University	138 (45.4%)
	Master’s/Doctorate	36 (11.8%)
Psychiatric support	Yes	28 (9.2%)
	No	276 (90.8%)
Time since loss	M (SD)	13.91 (5.77)
Emotional closeness	M (SD)	7.31 (2.38)
Perceived social support	M (SD)	6.01 (2.61)
Relationship to deceased	First-degree relative	66 (21.7%)
	Second-degree relative	98 (32.2%)
	Third-degree relative	92 (30.3%)
	Friend	41 (13.5%)
	Spouse	7 (2.3%)
Cause of death	Illness	255 (83.9%)
	Accident	33 (10.9%)
	Homicide	11 (3.6%)
	Suicide	5 (1.6%)
Frequency of contact	Every day	55 (18.1%)
	More than once a week	66 (21.7%)
	More than once a month	81 (26.6%)
	More than twice a year	59 (19.4%)
	Once a year or less	43 (14.1%)
Another loss	Yes	67 (22.0%)
	No	237 (78.0%)
Sudden loss	Yes	105 (34.5%)
	No	199 (65.5%)

Note. N = 304. Values are n (%) unless otherwise indicated. M = mean; SD = standard deviation.

**Table 2:** Descriptive Statistics for Study Variables.

Scale	Subscale	M	SD	Min	Max
Grief Scale	Physical	10.47	4.55	5	25
	Emotional	20.80	5.87	10	37
	Cognitive	27.02	5.40	13	44
	Behavioral	18.04	7.57	10	46
	Total grief	76.34	19.88	43	139
Cognitive Emotion Regulation	Self-blame	8.76	3.55	4	20
	Acceptance	14.18	3.55	4	20
	Rumination	12.84	3.69	4	20
	Positive refocusing	12.72	3.02	4	20
	Refocus on planning	13.92	3.59	4	20
	Positive reappraisal	14.20	3.62	4	20
	Putting into perspective	12.73	3.37	4	20
	Catastrophizing	9.12	3.42	4	20
	Blaming others	8.50	3.40	4	20
	Total	107.12	18.60	36	155
Intolerance of Uncertainty	Uncertainty is stressful and upsetting	28.71	8.77	9	53
	Negative self-assessments about uncertainty	22.01	6.84	8	39
	Disturbing thoughts about the uncertainty of the future	12.19	3.98	4	20
	Uncertainty interferes with action	12.15	3.82	4	20
	Total	78.45	22.01	26	128

**Table 3:** Grief Reactions According to Demographic Characteristics.

Grief Dimension	Characteristic/Group	n	M	SD	Statistic	Raw *p*	Holm-Adjusted *p*	Effect Size
Physical	Gender: Women	204	10.60	4.63	t(302) = 0.733	0.464	1.000	g = 0.09 [−0.15, 0.33]
	Gender: Men	100	10.20	4.41				
Emotional	Gender: Women	204	20.89	5.88	t(302) = 0.379	0.705	1.000	g = 0.05 [−0.19, 0.28]
	Gender: Men	100	20.62	5.89				
Cognitive	Gender: Women	204	27.56	5.49	t(302) = 2.522	0.012	0.049	g = 0.31 [0.07, 0.55]
	Gender: Men	100	25.92	5.05				
Behavioral	Gender: Women	204	17.90	7.92	t(302) = −0.466	0.642	1.000	g = −0.06 [−0.29, 0.18]
	Gender: Men	100	18.32	6.82				
Physical	Economic status: Low	56	11.25	5.14	F(2, 301) = 1.086	0.339	0.757	ω^2^ = 0.001
	Economic status: Moderate	171	10.21	4.45				
	Economic status: High	77	10.48	4.31				
Emotional	Economic status: Low	56	20.94	5.82	F(2, 301) = 1.674	0.189	0.757	ω^2^ = 0.004
	Economic status: Moderate	171	20.31	5.71				
	Economic status: High	77	21.77	6.21				
Cognitive	Economic status: Low	56	27.41	4.47	F(2, 301) = 0.475	0.622	0.757	ω^2^ = 0.000
	Economic status: Moderate	171	26.76	5.45				
	Economic status: High	77	27.33	5.91				
Behavioral	Economic status: Low	56	19.58	7.58	F(2, 301) = 1.512	0.222	0.757	ω^2^ = 0.003
	Economic status: Moderate	171	17.56	7.38				
	Economic status: High	77	17.97	7.93				
Physical	Education: Primary school	10	12.80	7.58	F(4, 299) = 1.378	0.241	0.724	ω^2^ = 0.005
	Education: Secondary school	18	11.44	5.55				
	Education: High school	102	10.01	4.28				
	Education: University	138	10.32	4.33				
	Education: Master’s/Doctorate	36	11.19	4.49				
Emotional	Education: Primary school	10	21.10	6.78	F(4, 299) = 0.929	0.447	0.895	ω^2^ = 0.000
	Education: Secondary school	18	21.33	6.80				
	Education: High school	102	20.08	5.85				
	Education: University	138	20.87	5.69				
	Education: Master’s/Doctorate	36	22.19	5.92				
Cognitive	Education: Primary school	10	29.80	7.08	F(4, 299) = 0.796	0.529	0.895	ω^2^ = 0.000
	Education: Secondary school	18	26.55	5.17				
	Education: High school	102	26.69	5.45				
	Education: University	138	27.10	5.12				
	Education: Master’s/Doctorate	36	27.11	5.95				
Behavioral	Education: Primary school	10	21.50	11.86	F(4, 299) = 1.732	0.143	0.571	ω^2^ = 0.010
	Education: Secondary school	18	20.61	7.51				
	Education: High school	102	17.00	6.85				
	Education: University	138	17.94	7.58				
	Education: Master’s/Doctorate	36	19.13	7.81				

Note. M = mean; SD = standard deviation; CI = confidence interval. Hedges’ g with 95% confidence intervals is reported for gender comparisons; ω^2^ is reported for multi-group one-way ANOVAs. Negative ω^2^ estimates were truncated to 0.000 for reporting. Raw *p* values are unadjusted. Holm-adjusted *p* values were calculated separately within each demographic characteristic across the four grief dimensions.

**Table 4:** Comparison of Grief Subscale Scores by Degree of Kinship and Cause of Death.

Grief	Variable	Group	n	M	SD	Welch F	Raw *p*	Holm *p*	ω^2^
Physical	Degree of kinship	First-degree relative	66	13.36	4.49	17.98	<0.001	<0.001	0.213
		Second-degree relative	98	10.89	3.94				
		Third-degree relative	92	7.73	3.22				
		Friend	41	10.04	4.85				
		Spouse	7	15.71	4.38				
Emotional	Degree of kinship	First-degree relative	66	24.16	5.77	9.95	<0.001	0.002	0.151
		Second-degree relative	98	20.81	4.83				
		Third-degree relative	92	17.52	4.56				
		Friend	41	21.75	7.18				
		Spouse	7	26.42	3.55				
Cognitive	Degree of kinship	First-degree relative	66	29.51	5.71	7.71	0.001	0.006	0.125
		Second-degree relative	98	27.34	5.21				
		Third-degree relative	92	24.45	4.55				
		Friend	41	27.36	4.76				
		Spouse	7	30.85	5.14				
Behavioral	Degree of kinship	First-degree relative	66	21.92	8.13	23.96	<0.001	<0.001	0.144
		Second-degree relative	98	17.81	7.08				
		Third-degree relative	92	14.46	5.49				
		Friend	41	18.65	7.65				
		Spouse	7	28.00	6.58				
Physical	Cause of death	Illness	255	10.37	4.38	0.53	0.751	1.000	0.000
		Accident	33	10.66	5.35				
		Homicide	11	12.63	5.83				
		Suicide	5	9.60	4.50				
Emotional	Cause of death	Illness	255	20.37	5.69	1.30	0.411	1.000	0.006
		Accident	33	23.06	6.54				
		Homicide	11	24.27	6.52				
		Suicide	5	20.20	4.14				
Cognitive	Cause of death	Illness	255	26.76	5.39	0.42	0.818	1.000	0.000
		Accident	33	28.12	4.86				
		Homicide	11	28.54	6.91				
		Suicide	5	29.60	5.07				
Behavioral	Cause of death	Illness	255	17.57	7.35	0.52	0.756	1.000	0.004
		Accident	33	20.12	7.63				
		Homicide	11	21.09	10.63				
		Suicide	5	21.40	8.50				

Note. M = mean; SD = standard deviation; ω^2^ = omega squared. Welch’s ANOVA was used because of unequal subgroup sizes and potential heterogeneity of variances. Holm-adjusted *p* values were calculated separately within each loss-related characteristic across the four grief dimensions. Significant kinship effects were followed by Games–Howell post hoc comparisons. Comparisons involving the spouse group and the smallest cause-of-death groups were considered exploratory and interpreted cautiously. No significant omnibus effect of cause of death was observed; therefore, post hoc comparisons were not interpreted for cause of death.

**Table 5:** Comparison of Grief Subscale Scores by Frequency of Contact, Psychiatric Support, Another Loss, and Sudden Loss.

Grief Variable	Group	n	M	SD	t/F	Raw *p*	Holm *p*	Effect Size [95% CI]
Physical	Frequency of contact: Every day	55	13.67	4.45	18.701	<0.001	<0.001	ω^2^ = 0.174
	More than once a week	66	10.83	4.16				
	More than once a month	81	10.83	4.27				
	More than twice a year	59	8.89	4.18				
	Once a year or less	43	7.30	3.29				
Emotional	Frequency of contact: Every day	55	24.30	5.30	13.147	<0.001	<0.001	ω^2^ = 0.125
	More than once a week	66	21.45	6.13				
	More than once a month	81	21.11	5.74				
	More than twice a year	59	18.86	4.73				
	Once a year or less	43	17.39	5.16				
Cognitive	Frequency of contact: Every day	55	29.92	5.24	18.787	<0.001	<0.001	ω^2^ = 0.151
	More than once a week	66	28.19	4.68				
	More than once a month	81	27.39	5.24				
	More than twice a year	59	25.52	5.41				
	Once a year or less	43	22.88	3.75				
Behavioral	Frequency of contact: Every day	55	22.43	8.27	12.324	<0.001	<0.001	ω^2^ = 0.114
	More than once a week	66	19.06	7.09				
	More than once a month	81	18.09	7.50				
	More than twice a year	59	15.81	6.79				
	Once a year or less	43	13.81	4.99				
Physical	Psychiatric support: Yes	28	14.28	2.57	7.540	<0.001	<0.001	g = 0.95 [0.56, 1.35]
	No	276	10.08	4.53				
Emotional	Psychiatric support: Yes	28	28.07	3.60	10.568	<0.001	<0.001	g = 1.48 [1.07, 1.88]
	No	276	20.06	5.55				
Cognitive	Psychiatric support: Yes	28	31.00	3.69	5.695	<0.001	<0.001	g = 0.83 [0.44, 1.22]
	No	276	26.62	5.39				
Behavioral	Psychiatric support: Yes	28	21.92	4.44	4.462	<0.001	<0.001	g = 0.57 [0.18, 0.96]
	No	276	17.64	7.72				
Physical	Another loss: Yes	67	12.49	4.11	4.453	<0.001	<0.001	g = 0.58 [0.31, 0.86]
	No	237	9.90	4.52				
Emotional	Another loss: Yes	67	22.58	5.76	2.867	0.005	0.015	g = 0.39 [0.12, 0.67]
	No	237	20.29	5.82				
Cognitive	Another loss: Yes	67	28.50	5.50	2.516	0.013	0.019	g = 0.35 [0.08, 0.63]
	No	237	26.60	5.31				
Behavioral	Another loss: Yes	67	20.19	7.57	2.642	0.010	0.019	g = 0.37 [0.09, 0.64]
	No	237	17.43	7.48				
Physical	Sudden loss: Yes	105	11.43	5.10	2.541	0.012	0.012	g = 0.33 [0.09, 0.56]
	No	199	9.96	4.16				
Emotional	Sudden loss: Yes	105	22.25	6.05	3.113	0.002	0.006	g = 0.38 [0.14, 0.62]
	No	199	20.03	5.64				
Cognitive	Sudden loss: Yes	105	28.99	5.58	4.630	<0.001	<0.001	g = 0.58 [0.34, 0.82]
	No	199	25.98	5.01				
Behavioral	Sudden loss: Yes	105	19.90	8.94	2.873	0.005	0.009	g = 0.38 [0.14, 0.62]
	No	199	17.06	6.56				

Note. M = mean; SD = standard deviation. Welch’s ANOVA was used for frequency-of-contact comparisons; ω^2^ is reported as the effect-size estimate. Welch’s independent-samples *t* test was used for two-group comparisons, with Hedges’ g and 95% confidence intervals reported as effect sizes. Raw *p* values are unadjusted; Holm-adjusted *p* values were calculated separately within each loss-related characteristic across the four grief dimensions.

**Table 6:** Correlations of Grief with Age, Time Since Loss, Emotional Closeness, and Perceived Social Support.

Variable	r	95% CI	Raw *p*	Holm *p*
Age	−0.012	[−0.124, 0.101]	0.835	0.835
Time since loss	−0.053	[−0.165, 0.060]	0.357	0.714
Emotional closeness	0.549	[0.465, 0.623]	<0.001	<0.001
Perceived social support	0.202	[0.092, 0.308]	<0.001	0.001

Note. Pearson correlations with total grief. CI = confidence interval. Conventional two-sided 95% CIs were calculated using Fisher’s r-to-z transformation. Holm adjustment was applied across the four correlations. Statistics are based on N = 304.

**Table 7:** Correlations Between Grief and Cognitive Emotion Regulation Strategies.

Variable	r	95% CI	Raw *p*	Holm *p*
Self-blame	0.445	[0.350, 0.531]	<0.001	<0.001
Acceptance	−0.162	[−0.270, −0.050]	0.005	0.019
Rumination	0.157	[0.045, 0.265]	0.006	0.019
Positive refocusing	−0.192	[−0.298, −0.081]	<0.001	0.005
Refocus on planning	−0.148	[−0.256, −0.036]	0.010	0.020
Positive reappraisal	−0.192	[−0.298, −0.081]	<0.001	0.005
Putting into perspective	0.044	[−0.069, 0.156]	0.445	0.445
Catastrophizing	0.532	[0.446, 0.608]	<0.001	<0.001
Blaming others	0.348	[0.245, 0.443]	<0.001	<0.001
Cognitive emotion regulation total	0.152	[0.040, 0.260]	0.008	—

Note. Pearson correlations with total grief. CI = confidence interval. Conventional two-sided 95% CIs were calculated using Fisher’s r-to-z transformation. Holm adjustment was applied across the nine individual cognitive emotion regulation strategies. The total CER score is shown descriptively and was not included in that nine-test Holm family. Statistics are based on N = 304.

**Table 8:** Correlations Between Grief and Intolerance of Uncertainty.

Variable	r	95% CI	Raw *p*	Holm *p*
Uncertainty is stressful and upsetting	0.380	[0.279, 0.472]	<0.001	<0.001
Negative self-assessments about uncertainty	0.447	[0.352, 0.533]	<0.001	<0.001
Disturbing thoughts about the uncertainty of the future	0.434	[0.338, 0.521]	<0.001	<0.001
Uncertainty interferes with action	0.359	[0.257, 0.453]	<0.001	<0.001
Intolerance of uncertainty total	0.447	[0.352, 0.533]	<0.001	—

Note. Pearson correlations with total grief. CI = confidence interval. Conventional two-sided 95% CIs were calculated using Fisher’s r-to-z transformation. Holm adjustment was applied across the four intolerance-of-uncertainty dimensions. The total score is shown descriptively and was not included in that four-test Holm family. Statistics are based on n = 304.

**Table 9:** Hierarchical Multiple Regression Analysis Examining Incremental Associations with Grief.

Panel A. Model Summary							
**Block**	**Variables Added**	***R*** **^2^**	**Adjusted *R*^2^**	**Δ*R*^2^**	**ΔF**	**F**	***p***
1	Demographic and loss-related covariates	0.447	0.400	—	—	9.53	<0.001
2	Adaptive and maladaptive CER	0.629	0.593	0.182	42.97	17.45	<0.001
3	Intolerance of uncertainty	0.640	0.603	0.011	5.48	17.21	0.020
Panel B. Final Model Coefficients (HC3 Robust Standard Errors)							
**Predictor**		**B**	**Robust SE**	**β**	**t**	***p***	**95% CI for B**
Intercept		53.162	11.169	—	4.76	<0.001	[31.118, 75.205]
Age		−0.044	0.098	−0.026	−0.45	0.656	[−0.237, 0.150]
Gender: Women (ref. Men)		−0.073	2.207	−0.002	−0.03	0.974	[−4.428, 4.282]
Emotional closeness		3.638	0.749	0.393	4.85	<0.001	[2.159, 5.117]
Perceived social support		−0.257	0.495	−0.031	−0.52	0.603	[−1.234, 0.719]
Sudden loss: No (ref. Yes)		−4.559	2.125	−0.105	−2.15	0.033	[−8.754, −0.365]
Another loss: No (ref. Yes)		−2.052	2.833	−0.042	−0.72	0.470	[−7.643, 3.539]
Psychiatric support: No (ref. Yes)		−3.578	2.690	−0.070	−1.33	0.185	[−8.888, 1.733]
Contact: More than once a week (ref. Every day)		−2.409	3.848	−0.046	−0.63	0.532	[−10.005, 5.186]
Contact: More than once a month (ref. Every day)		0.498	3.721	0.011	0.13	0.894	[−6.847, 7.842]
Contact: More than twice a year (ref. Every day)		−7.178	3.993	−0.146	−1.80	0.074	[−15.059, 0.702]
Contact: Once a year or less (ref. Every day)		0.975	4.447	0.017	0.22	0.827	[−7.802, 9.752]
Kinship: Second-degree relative (ref. First-degree relative)		−2.756	3.250	−0.065	−0.85	0.398	[−9.170, 3.659]
Kinship: Third-degree relative (ref. First-degree relative)		−7.213	3.136	−0.156	−2.30	0.023	[−13.403, −1.024]
Kinship: Friend (ref. First-degree relative)		−0.394	4.812	−0.007	−0.08	0.935	[−9.891, 9.104]
Kinship: Spouse (ref. First-degree relative)		11.170	6.905	0.068	1.62	0.108	[−2.459, 24.798]
Adaptive CER		−0.380	0.088	−0.252	−4.31	<0.001	[−0.554, −0.206]
Maladaptive CER		0.666	0.148	0.350	4.49	<0.001	[0.373, 0.958]
IU total		0.146	0.066	0.145	2.21	0.029	[0.015, 0.276]

Note. Block 1 included age, gender, emotional closeness to the deceased, perceived social support, sudden loss, experience of another loss, psychiatric support, frequency-of-contact dummy variables, and degree-of-kinship dummy variables. Reference categories are indicated explicitly in Panel B. For binary loss-related variables, “Yes” served as the reference category. For frequency of contact, “Every day” served as the reference category; for degree of kinship, “First-degree relative” served as the reference category; and for gender, men served as the reference category. CER = cognitive emotion regulation; IU = intolerance of uncertainty; CI = confidence interval; Δ*R*^2^ = change in explained variance; ΔF = F change. HC3 robust standard errors and 95% confidence intervals are reported for the final model coefficients.

## Data Availability

The anonymized data supporting the findings of this study are available from the corresponding author upon reasonable request.
